# 以突眼、视力减退、腹膜后纤维化为表现的Erdheim-Chester病一例报告并文献复习

**DOI:** 10.3760/cma.j.issn.0253-2727.2021.08.010

**Published:** 2021-08

**Authors:** 天骅 何, 艾琳 赵, 娜 牛, 凤丹 王, 杰 师, 剑 李, 欣欣 曹

**Affiliations:** 1 中国医学科学院、北京协和医学院北京协和医院血液内科 100730 Department of Hematology, Peking Union Medical College Hospital, Chinese Academy of Medical Sciences and Peking Union Medical College, Beijing 100730, China; 2 四川大学华西医院血液内科，成都 611130 West China Hospital, Sichuan University, Chengdu 611130, China; 3 中国医学科学院、北京协和医学院北京协和医院核医学科 100730 Department of Nuclear Medicine, Peking Union Medical College Hospital, Chinese Academy of Medical Sciences and Peking Union Medical College, Beijing 100730, China; 4 中国医学科学院、北京协和医学院北京协和医院放射科 100730 Department of Radiology, Peking Union Medical College Hospital, Chinese Academy of Medical Sciences and Peking Union Medical College, Beijing 100730, China; 5 中国医学科学院、北京协和医学院北京协和医院病理科 100730 Department of Pathology, Peking Union Medical College Hospital, Chinese Academy of Medical Sciences and Peking Union Medical College, Beijing 100730, China

## 病例资料

患者，男，52岁，因“双眼突出、视力减退2年”在2017年就诊于北京协和医院。患者2015年无明显诱因出现双眼睑皮下肿物，眼球突出，无眼痛，局部皮肤无红肿破溃。患者症状进行性加重伴视力下降，乏力，下肢凹陷性水肿。患者2016年11月就诊于当地医院，诊断“炎性假瘤”，接受泼尼松60 mg每日1次口服治疗，突眼、乏力、水肿缓解。泼尼松减量至20 mg每日1次后症状复发。患者于2017年3月就诊于我院，查血常规示WBC 21.59×10^9^/L，ANC 15.31×10^9^/L，HGB 136 g/L，PLT 453×10^9^/L；肝肾功能正常；红细胞沉降率（ESR）41 mm/1 h，超敏C反应蛋白（hsCRP）56.51 mg/L；补体、IgG正常；抗核抗体（ANA）及抗中性粒细胞胞浆抗体（ANCA）阴性。2017年3月，患者眼眶增强MRI示双侧球后脂肪间隙内团块状等T1等T2信号影，形态不规则，增强后强化明显。左蝶窦及双侧上颌窦内异常信号填充，性质同球后病变。胸腹部CT示胸主动脉下段、腹主动脉周围软组织密度影包绕；双侧肾盂肾盏积水、肾周脂肪浑浊；双肺多发结节斑片影，双侧胸膜增厚；心包积液；胰腺脂肪浸润；双侧肱骨头、锁骨及部分胸椎、肋骨内斑片状高密度影。患者既往体健，否认有毒有害物质接触或长期药物使用史，个人史、家族史无特殊。诊断“坏死性肉芽肿性多血管炎（GPA），继发/合并腹膜后纤维化（RPF）”，予泼尼松60 mg口服每日1次，环磷酰胺100 mg每日1次治疗。患者突眼、乏力症状稍缓解，复查ESR 11～16 mm/1 h，hsCRP 10.81～17.29 mg/L；ANCA（−）；眼眶MRI示双侧眶内、海绵窦占位范围基本同前；胸腹CT示主动脉周围软组织密度影、肾周索条及絮状影、双侧肾盂积水、多发椎体及骨盆高密度影，基本同前。2018年1月患者治疗效果不佳，多发骨硬化改变不除外转移瘤，完善肿瘤标志物未见明显升高，骨髓涂片大致正常；PETCT：胸主动脉下段及腹主动脉上段血管旁软组织密度影，代谢不均匀增高（SUVmax 2.8）；双肾增大，肾周索条及絮状影；全身骨多发高密度影，部分代谢增高（左髂嵴SUVmax 1.7）；双肺多发小结节，其中右肺尖小结节代谢稍高（SUVmax 0.8），双侧胸膜稍增厚；右侧上颌窦软组织密度影伴代谢增高（SUVmax 10.2）；右房周围摄取增高（SUVmax 3.8）。继续激素规律减量治疗，调整环磷酰胺为0.6～1 g，静脉输注，每周1次，并先后加用吗替麦考酚酯、甲氨蝶呤、他克莫司等。2018年起加用他莫昔芬治疗腹膜后纤维化。

2019年5月患者再次复查CT示主动脉、腹膜后、肺及胸膜病变同前；眼眶MRI示病变范围较前略增加。考虑患者多系统受累，包括鼻窦、眶周、大血管、肾周、输尿管、骨骼、肺等，激素及免疫抑制剂治疗效果不佳，GPA合并RPF不足以解释全部病情。再次评价骨骼病变，骨扫描示双侧眼眶骨、股骨、胫骨、额骨、上颌骨异常，提示Erdheim-Chester病（ECD）。进一步完善相关检查：hsCRP 11.38 mg/L；IL-6 21 pg/ml（正常参考值<5.9 pg/ml），IL-8、IL-10、TNF-α正常，ESR 60 mm/1h。头增强MRI：双侧小脑幕下脑外多发类圆形异常等T1等T2信号，较大者约1.7 cm×1.2 cm，略不均匀强化。促甲状腺激素、促肾上腺皮质激素、性激素等均正常。心肌灌注延迟成像动态MRI：右心房周围、右房室沟内及主动脉根部周围团片样异常信号。患者2019年7月行右眼睑肿物活检术，病理示纤维组织中可见泡沫样富含脂质的组织细胞浸润，散在Touton型多核巨细胞（图1A），免疫组化为CD1a（−）（图1B），BRAF（部分+）（图1C），CD68（+）（图1D），Cyclin D1（部分+），S-100（−）。组织基因测序检测到BRAF p.V600E突变。结合病史、影像学检查及病理，考虑ECD诊断明确。停用免疫抑制剂及他莫昔芬，因患者存在BRAF V600E突变，予口服维莫非尼480 mg每日2次。3个月后随诊，hsCRP 20.46 mg/L，ESR 29 mm/1h，IL-6 5.4 pg/ml，余IL-8、IL-10、TNF-α正常。患者突眼较前明显缓解，眼睑黄瘤明显缩小，乏力、水肿明显改善。2020年2月患者hsCRP 5.64 mg/L，IL-6 5.8 pg/ml。胸腹盆增强CT示主动脉、肾周、肾盂及输尿管等病变较前未见明显变化；双肺散在小结节，部分较前变淡。PET-CT示主动脉周围病变基本同前（SUVmax 1.8），心脏异常摄取增高灶本次未见，上颌窦病变放射性摄取减低（SUVmax 0.7），骨多发高密度影基本同前（左髂嵴SUVmax 1.4）。维莫非尼治疗过程中，患者出现皮肤瘙痒、光过敏、脱发、肩关节痛等不良反应，对症治疗后耐受良好。

**图1:**
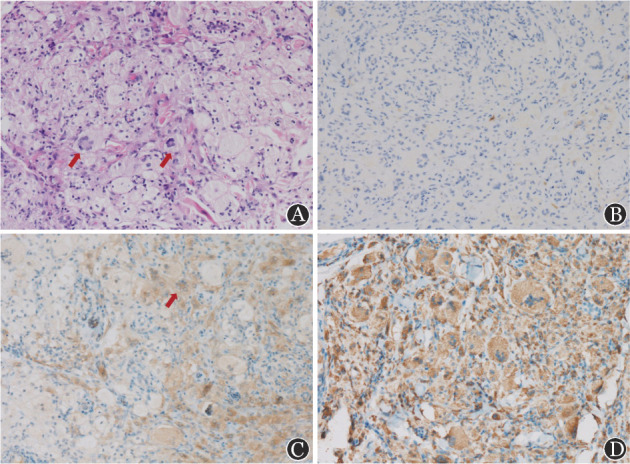
患者右眼睑肿物活检病理HE染色及免疫组化染色 A：HE染色（×100），可见Touton型多核巨细胞（箭头所指）；B：CD1a免疫组化染色（×100）；C：BRAF免疫组化染色（×100），可见Touton型多核巨细胞（箭头所指）；D：CD68免疫组化染色（×100）

## 讨论及文献复习

ECD是一种罕见的非朗格汉斯细胞组织细胞疾病，自1930年报道第一例以来文献共记载约1500例，其诊断依赖典型的临床表现和组织病理表现，并除外其他诊断[1]–[2]。ECD可累及全身几乎所有系统（表1），但受累的各系统往往缺乏相应临床症状[11]。本例患者以球后肿物起病，存在眼睑黄瘤、鼻窦软组织占位、腹膜后纤维化、多发骨硬化、主动脉周围纤维化、右心房周围软组织浸润、硬脑膜占位、肺内多发结节斑片影，均符合ECD典型表现。ECD的典型病理表现为泡沫样组织细胞浸润，夹杂炎症细胞和多核巨细胞（Touton细胞）浸润，并纤维组织混合其中或包绕其外。ECD细胞CD68（+）、CD163（+）、FXⅢa（+），S-100（+/−），但不同于朗格汉斯细胞组织细胞增生症（LCH），其CD1a（−）。

**表1:** Erdheim-Chester病各系统/器官典型临床、影像学表现及发病率[2]–[10]

系统/器官（发病率）	临床及影像学表现
骨骼（80％～95％）	长骨骨干、干骺端对称性骨硬化，颅面骨骨硬化；核素骨扫描可见典型四肢骨干骺端双侧对称性放射浓聚
鼻窦（59％）	鼻窦内软组织浸润
大血管（40％～59％）	主动脉周围纤维化，可累及胸主动脉、腹主动脉及其一级分支
腹膜后（33％～59％）	腹膜后纤维化所致输尿管梗阻、肾盂扩张；肾周脂肪浸润
心脏（49％～57％）	右心浸润/假瘤；心包增厚/积液；冠状动脉周围浸润、心肌梗死；瓣膜病变；心脏传导异常
肺（30％～50％）	肺实质受累表现为多发结节、磨玻璃影、实变、小叶间隔增厚、叶间裂增厚等；胸膜受累表现为胸膜增厚；肺功能一般无明显异常
中枢神经系统（约40％）	临床症状多样，包括锥系症状、锥体外系症状、精神症状等。影像学表现为颅内占位病变，硬脑膜增厚或肿块，颅内血管浸润；垂体受累表现为尿崩症、垂体前叶功能异常（生长激素缺乏、促性腺激素缺乏、促甲状腺激素缺乏、高泌乳素血症）
眼眶（22％～25％）	球后软组织浸润，临床表现为突眼、视力损害
皮肤（22％～27％）	皮肤黄瘤，常见于眼睑、眶周
其他少见部位	齿龈及牙槽骨病变、胃肠道及胆系受累，另有病例报道甲状腺、乳腺、睾丸等受累

ECD发病率低，各系统临床表现多样，一直是诊断和鉴别诊断的难点。本例曾一度被误诊为GPA，但患者存在GPA难以解释的多发骨硬化灶，核素骨扫描可见典型四肢骨干骺端双侧对称性放射浓聚，这一特征性临床表现为修正诊断提供了重要线索。此外，GPA与ECD的鉴别还可从以下几个方面进行：①GPA患者多系统受累常包括眼、耳、鼻窦、肺和肾，而不包括骨骼、大血管、腹膜后等ECD常见的病变部位[12]。②GPA的眼部病变以结膜炎、角膜炎、巩膜炎、视网膜血管炎、葡萄膜炎、视神经病变等更为常见，眶内假瘤也可出现[13]–[14]。ECD常见眶内假瘤，眼部其他结构炎症表现不明显。③GPA的耳、鼻窦受累程度较重，伴局部软骨破坏，出现脓性/血性鼻分泌物、鞍鼻畸形、耳漏等[12]。ECD的鼻窦受累以浸润和软组织占位为主，局部结构破坏不明显。④GPA常见肺部多发实性结节、斑片影，患者往往有明显的咳嗽、喘息、咯血等症状，另可有大气道受累[15]。而ECD的肺部病变多为多发实性/磨玻璃小结节、胸膜及小叶间隔增厚，患者往往无呼吸系统症状，肺功能基本保留。⑤GPA的肾脏受累为寡免疫复合物型肾小球肾炎，临床肾功能受损常见[12]，与ECD患者肾周浸润、肾盂及输尿管周围浸润、肾盂扩张等表现迥异。⑥>80％的GPA患者ANCA阳性[12]，未见ECD与ANCA相关的报道。

除GPA以外，ECD也常被误诊为IgG4相关疾病（IgG4-related disease，IgG4-RD）。IgG4-RD可有眼眶假瘤表现，但其他常见临床表现包括淋巴结炎、硬化性胆管炎、泪腺/涎腺肿大、间质性肾炎、血清IgG4水平升高等在ECD中均未见报道[16]–[17]。IgG4-RD也可导致RPF，常见腹主动脉、髂动脉旁纤维化，继发下输尿管梗阻多见，不同于ECD常见的以肾周、肾盂、上输尿管周围为著的纤维化。GPA偶尔可合并IgG4-RD[18]，其临床表现更难以与ECD鉴别。

目前ECD病因不明。38％～100％的ECD患者存在BRAF V600E突变[19]–[20]，这一基因突变可激活RAS-RAF-MEK-ERK细胞信号通路，影响多种细胞功能包括细胞增殖、凋亡、血管生成和迁移等，迄今已在多种恶性肿瘤中发现高频BRAF V600E突变。结合ECD病理免疫组化分析中仅有泡沫样组织细胞和Touton巨细胞存在该突变，提示ECD本质上可能为一种克隆性疾病[20]。此外，有研究表明BRAF突变状态与ECD的心脏、大血管、心包、中枢神经系统受累相关[21]–[22]。这类泡沫样组织细胞可释放多种细胞因子和炎症因子，导致局部炎症细胞聚集和纤维结缔组织增生，并有相应的影像学表现。此外，组织细胞疾病中，BRAF V600E突变仅见于LCH和ECD，因而有助于鉴别其他疾病如窦组织细胞疾病（Rosai-Dorfman Disease，RDD）、幼年黄色肉芽肿等[23]。

已明确干扰素-α（IFN-α）治疗使患者生存获益，其作为一线治疗的有效率约80％，3年无进展生存率和总生存率分别为64％和84.5％[24]。BRAF抑制剂自2012年首次应用于ECD治疗以来，使存在相应BRAF V600E突变的难治、复发患者获得了显著的临床/影像学改善和持续的临床缓解[2]。目前维莫非尼已被美国FDA批准用于存在BRAF突变的ECD患者的一线治疗[20]。IFN-α治疗失败且无BRAF突变患者可采用全身性化疗，包括克拉屈滨或阿糖胞苷[25]–[26]。糖皮质激素治疗可短暂缓解症状，但缺乏长期治疗有效的证据。本例患者受累系统多，包括重要脏器如心脏、中枢神经系统，且存在BRAF突变，一线采用维莫非尼治疗，经过1年随访，该患者临床症状改善，影像学异常表现部分缓解。

综上，ECD为罕见的组织细胞疾病，可累及全身几乎所有器官和系统，诊断难度较大，如仅依靠局部临床表现往往容易误诊。本例患者按照GPA治疗两年疗效欠佳，存在系统性血管炎难以解释的多发骨硬化改变，重新整理思路、完善检查、获取病理后确诊为ECD。因此在常见病治疗效果不佳或存在难以解释的临床表现时，应当重新获取病史、开阔思路、审慎鉴别，或可别出机杼、柳暗花明。
